# Bioactive Glass Applications: A Literature Review of Human Clinical Trials

**DOI:** 10.3390/ma14185440

**Published:** 2021-09-20

**Authors:** Maria Cannio, Devis Bellucci, Judith A. Roether, Dino. N. Boccaccini, Valeria Cannillo

**Affiliations:** 1 Dipartimento di Ingegneria Enzo Ferrari, Università degli Studi di Modena e Reggio Emilia, Via P. Vivarelli 10, 41125 Modena, Italy; maria.cannio@unimore.it (M.C.); devis.bellucci@unimore.it (D.B.); 2 Department of Materials Science and Engineering, Institute for Polymer Materials, University of Erlangen-Nuremberg, 91058 Erlangen, Germany; judith.roether@fau.de; 3 Tecno Italia SRL, Via Emilia Romagna 83, 41049 Sassuolo, Italy; dino.boccaccini@hotmail.com

**Keywords:** clinical trials, Bioglass^®^ 45S5, BonAlive^®^, 13-93B3 bioactive glass, powders, monolithic material, scaffold, regeneration, implant, bioactivity, biologic response

## Abstract

The use of bioactive glasses in dentistry, reconstructive surgery, and in the treatment of infections can be considered broadly beneficial based on the emerging literature about the potential bioactivity and biocompatibility of these materials, particularly with reference to Bioglass^®^ 45S5, BonAlive^®^ and 19-93B3 bioactive glasses. Several investigations have been performed (i) to obtain bioactive glasses in different forms, such as bulk materials, powders, composites, and porous scaffolds and (ii) to investigate their possible applications in the biomedical field. Although in vivo studies in animals provide us with an initial insight into the biological performance of these systems and represent an unavoidable phase to be performed before clinical trials, only clinical studies can demonstrate the behavior of these materials in the complex physiological human environment. This paper aims to carefully review the main published investigations dealing with clinical trials in order to better understand the performance of bioactive glasses, evaluate challenges, and provide an essential source of information for the tailoring of their design in future applications. Finally, the paper highlights the need for further research and for specific studies intended to assess the effect of some specific dissolution products from bioactive glasses, focusing on their osteogenic and angiogenic potential.

## 1. Introduction

Biomaterials, natural or synthetic, are used in regenerative medicine, dentistry, and in the treatment of infections in a broad range of applications to replace damaged tissues or to restore biological functions [1,2,3,4,5,6,7]. A material could be an optimal biomaterial usable for clinical applications if it has the following properties: (1) it is available at any time and in any amount; (2) it is non-expensive; (3) it is inert so that it prevents body reactions or infections when implanted; (4) it is not toxic; (5) it could be easily shaped or molded during the operation; (6) it does not require any additional surgery time; (7) it does not disturb medical follow-up procedures during tests (e.g., computed tomography, magnetic resonance imaging, etc.). In this context, bioactive glasses (BG), originally developed by Hench starting in 1969, are considered a group of reactive materials with attractive properties, not only in terms of biocompatibility but also of bioactivity; this means that they show the ability to form bonds with mineralized bone tissue in the physiological body environment [8,9,10,11,12]. Most of them are based on the Na_2_O, CaO, P_2_O_5_, and SiO_2_ system—the same system as Hench’s original formulation—and have a weight percent of SiO_2_ < 55%. A higher amount of SiO_2_ will result in a loss of bioactivity. By combining/altering all the main components, such as sodium dioxide, calcium oxide, and phosphorous, different bioactive glasses can be developed, showing composition-dependent promising properties such as bone-forming capability, antibacterial properties, degradability, and even soft tissue regeneration and wound healing [2,5,8,13,14,15,16,17]. Bioactive glasses can be used in suitable solid form/shape (bulk and porous scaffolds) or in the form of powders for the deposition of coatings on biomedical devices or as composites (acting as fillers) [18]. Firstly, these materials were used in the form of solid shapes for the replacement of bone in middle ear prostheses [19]; then, many other different applications were proposed, including dentistry [7,20], tissue engineering [4,21], and bone regeneration medicine [11,22,23]. Another promising application is the use of bioactive glass loaded with antibiotics or drugs, which, as consequence of their degradation activity, are released during the treatment of bone infections [24,25]. These kinds of biomaterials have different degradation kinetics, which depend on the glass composition, the manufacturing route (such as sol–gel or classical melting route), the grade of porosity, and/or the presence of dopant ions such as silver, copper, strontium, and zinc [26,27]. The purpose of this review is to deliver a literature overview of the clinical results and conclusions about different applications of bioactive glasses, with a special emphasis on three main compositions developed in the last few decades: the standard silicate glass (45S5 or Bioglass^®^), a glass–ceramic (S53P4 bioactive glass or BonAlive^®^), and a borate-based glass (13-93B3 bioactive glass) [9,10,13,17,19,28,29,30], which appears particularly promising in wound healing. In particular, a specific section provides a comprehensive overview regarding the development of such compositions, their properties and mechanisms of action, and the main manufacturing techniques for the realization of products with different shapes, with a special regard to their application. Subsequently, applications and clinical trials involving the three considered main compositions are reported and discussed in detail.

## 2. Bioactive Glasses

### 2.1. Compositions

Since 1969, several types of bioactive glasses have been developed, such as the standard 45S5 silicate glass (named 45S5 or Bioglass^®^), antibacterial bioactive glasses (e.g., S53P4 or BonAlive^®^), and borate-based glasses (13-93B3 bioactive glass) [31]. In particular, the reference glass 45S5 Bioglass^®^ (45S5) is nowadays widely used in the treatment of periodontal osseous defects, spinal fusion, cranial and maxillo-facial reconstruction, and in the production of middle ear prostheses. Moreover, 45S5 can be considered the “parent” glass of several compositions, which have been obtained by adding or removing specific ions from the original formulation; some of these compositions are reported in Table 1. The 45S5 glass composition offers remarkable advantages due to its high bioactivity and osteoconductivity: during the dissolution of this BG, the release of its main ions (calcium, silica, sodium, and phosphate) occurs; then, they combine with the ions from the environment to form a carbonated hydroxyapatite (HCA) bone-like mineral coating. Finally, the HCA layer establishes tenacious bonds with the local surrounding bone, encouraging and stimulating its growth. In addition, many studies on 45S5 bioglass, both in vitro and in vivo, have shown that this glass can be used to produce scaffolds (at a tailored porosity exceeding 90%) for tissue engineering; thus, 45S5 can be considered a promising scaffold material. Scaffolds represent important components of tissue engineering, the biomedical engineering discipline that aims to develop biological substitutes to replace or repair failing tissues due to aging or specific pathologies. In fact, scaffolds are 3D structures that, thanks to their pores with a size from 100 μm to 300 μm, allow the penetration of cells, favor the secretion of vascular endothelial growth factor, and increase vascularization. On the other hand, scaffolds should have adequate mechanical strength to tolerate load-bearing applications and should be shaped and processed without breakage [32,33,34]. However, the high tendency of bioactive glasses to crystallize during thermal treatments, which are required for several manufacturing processes, such as the production of scaffolds (but also coatings and composites), has been the main obstacle to the broader diffusion of these materials in medical applications. In the last few years, some researchers have focused on the development of novel bioactive glasses with higher thermal stability (which means low tendency to devitrification) with respect to 45S5 [35,36,37,38,39].

S53P4 bioactive glass, commercially available as bioactive glass BonAlive^®^ (Biomaterials Ltd., Turku, Finland), is used as a bone graft filler in various orthopedical applications [40]. BonAlive^®^ was the first composition different from 45S5 to reach the market. Originally, it was investigated with the aim of extending the sintering range of bioactive glasses; as evident from its composition reported in Table 1, it has a higher silica content and this results in lower bioactivity with respect to 45S5 [31]. In fact, BonAlive^®^ has higher network connectivity and degrades more slowly. This material received European approval for applications as a bone graft substitute in orthopedic surgery in 2006. The published clinical results provide useful indications on S53P4′s ability to facilitate bone formation and bone defect healing and hence on its use in craniofacial surgery, grafting of bone defects after benign tumor resection, in instrumental spondylodesis, and in the treatment of tibial plateau fractures and of osteomyelitis [40]. S53P4 bioactive glass is usually used in granules with size ranging from 0.8 to 3.15 mm, or in the form of nonporous discs or plates with different shapes.

Recently, borate glasses have acquired great importance [4] due to their very encouraging results from pre-clinical or in vivo studies in the healing of chronic wounds (such as diabetic ulcers) with respect to conventional treatments [41,42]. In fact, the presence of boron guarantees the stimulation of vascularization and angiogenesis and an increase in the synthesis rate of RNA in fibroblasts [43]. A borate-based bioactive glass composition (13-93B3) is reported in Table 1, as well. This material, 13-93B3, is a borate glass, obtained from the 13-93 bioactive glass by substituting SiO_2_ with B_2_O_3_ and adding network modifiers such as K_2_O and MgO. The fact that 13-93B3 does not contain SiO_2_ allows faster degradation and a more complete conversion to HA with respect to 45S5 and S53P4 glasses, as explained in more detail in the following sections. In 2016, it was approved by the U.S. Food and Drug Administration as a suitable material to be used for wound healing [44].

The 13-93B3 glass has also been used in research applications to prepare scaffolds for tissue engineering [1,45,46]. In addition, based on in vivo studies, borate-based bioactive glasses could be considered of great interest for their ability to deliver antibiotics [47], even if additional investigations are required, since there is still a lack of clinical evidence.

**Table 1:** Chemical composition of 45S5 bioactive glass (Bioglass^®^), S53P4 bioactive glass (BonAlive^®^), and the 13-93B3 borate-based glass used for clinical trials in dentistry, reconstructive surgery (bone regeneration), and the treatment of infections [28,46].

Composition	45S5 Bioglass^®^ (wt%) [12]	S53P4 BonAlive^®^ (wt%) [48]	13-93B3 (wt%) [49]
Na_2_O	24.5	23	6
CaO	24.5	20	20
K_2_O	/	/	12
MgO	/	/	5
P_2_O_5_	6	4	4
SiO_2_	45	53	
B_2_O_3_	/	/	53

### 2.2. Properties of Bioactive Glasses and Mechanisms of Action

The biocompatibility of bioactive glasses depends on the silicate amount/concentration present in the glass: the optimum graft–bone bonding capability is reached when the concentration of silicate is in the range 45–52% [50]. Aspects of biocompatibility (cytotoxicity and genotoxicity), osteogenesis, and angiogenesis of different glasses have been investigated in order to identify the most promising bioactive glasses to be employed as possible substitutes in bone tissue regeneration and, more recently, in the treatment of infections. In particular, the literature suggests that the behavior of the main proteins (such as collagen, alkaline phosphatase, bone morphogenetic proteins (BMP2), transforming growth factor beta (TGF-β), fibroblast growth factors (FGFs)) involved in the process of the formation of new bone is determined by the ions present in the glass compositions [2,15,19,35]. The general mechanism of a bioglass used in medicine for bone regeneration or dental trauma can be explained as follows: after the implantation of a bioactive glass exposed to (body) fluid, some surface reactions occur that ensure the formation of a deposit of a calcium phosphate layer [20]. In fact, the release from the glass surface of significant concentrations of sodium, silica, calcium, and phosphate ions increases the local pH and osmotic pressure; then, a layer of silica gel covers the glass surface, and thereafter, amorphous calcium phosphates precipitate on it. The crystallization of these amorphous structures into hydroxyapatite seems to activate osteoblasts needed for the growth of new bone [26]. Due to the continuous reactions occurring on the glass surface and layer, the glass can be absorbed. In addition, these surface reactions are also responsible for the antibacterial properties of the glass and can potentially stimulate angiogenesis. As shown in the literature, the mechanism described above is based on two different steps: (1) the degradation of the bioactive glasses and (2) the release of specific ions from the glass. This mechanism is known as graft–bone bonding. The speed rate required to dissolve the bioactive glasses depends on their composition and can range from a few hours to some months.

The antibacterial effect of bioactive glasses such as S53P4 is attributed to the dissolution of alkaline ions from the surface [51,52]. As explained above, this process causes a rise in pH and osmotic pressure and it is proven to have bactericidal effects on different bacterial strains, such as *Staphylococcus aureus*, *Staphylococcus epidermidis*, *Escherichia coli*, and *Klebsiella pneumonia* [53,54].

In addition, mesoporous bioactive glass materials have been developed due to technical advances in BG processing to extend the potential of bioglass application into the field of design for innovative drug delivery systems. In this field, some bioactive glasses, different in composition if compared to common bioactive glasses, are used as antibiotic delivery devices [55]. In particular, these systems are mainly based on mesoporous bioactive glasses obtained using sol–gel methods, loaded with antibacterial agents or growth factors, characterized by a tailored and low rate of release to induce therapeutic effects (only 20–25% after 3 months) [56,57].

Borate bioactive glass, on the other hand, appears particularly promising when used as a carrier for antibiotic delivery, but further investigation is required since the evidence is not circumstantial [58,59]. As already mentioned, borate bioactive glasses show a faster degradation mechanism with respect to silicate-based glasses [13,46,60]. The more rapid healing rates demonstrated by these materials, when compared to silicate-based glasses, are mainly due to the high release rates of Ca^2+^ and (BO_3_)^3−^ ions. The release of PO_4_^3−^ in body fluids leads to the formation of an HCA directly on the unreacted glass [61]; however, in this case, no borate-rich layer forms on the glass surface, differently from silicate glasses, where a layer of silica slows down the formation of HA [45,62].

### 2.3. Manufacturing of Bioactive Glasses

Bioactive glasses can be produced using different manufacturing methods [63,64,65,66,67,68,69,70,71,72,73,74,75]. The most common methods are [76] melt quench synthesis and sol–gel.

#### 2.3.1. Melt Quench Synthesis

This is the traditional technology first used in 1969 by L. Hench. It includes as a first step melting at high temperatures, generally between 1300 °C and 1450 °C, in platinum crucibles (to avoid contamination), of different oxides, such as SiO_2_, Na_2_O, CaO, and P_2_O_5_; moreover, oxides of zinc, magnesium, titanium, boron, silver, etc., can be added to bioactive glasses to enhance the glass functionality and bioactivity. The second step is annealing, which consists in heating the produced bioglass to reduce the internal stresses due to the high thermal expansion of the material. In addition, annealing may reduce the amount of volatile alkali metal oxides and eventually favor the precipitation of apatite crystals in the glass matrix.

#### 2.3.2. The Sol–Gel Method

The method of sol–gel, first demonstrated in 1991 by Li et al. [70], requires lower temperatures (600–700 °C) than melt quench synthesis. First, a solution (sol) is prepared mixing metal–organic and metal salt used as precursors (i.e., tetraethyl orthosilicate, calcium nitrate, and triethylphosphate). Then, hydrolysis and condensation reactions occur and lead to the formation of a gel. Finally, a thermal treatment is required to dry the sol and make oxide formation and organic removal possible. This manufacturing process offers the advantage of better control of the composition and product homogeneity than traditional quenching methods [77]. Furthermore, sol–gel-type bioactive glasses have a greater surface area and higher porosity (mesoporous volume in mesoporous bioactive glasses) than melt-derived glasses having the same composition: glasses produced by sol–gel methods will be characterized by a higher degree of interaction with the surrounding environment and degradation, which results in more efficient bioactivity [63,69,78,79]. Nowadays, the preparation of mesoporous BG with high quality is still a challenge. Other methods that can be used in the manufacturing of bioactive glass to fabricate porous BG are additive manufacturing technologies [72]. Additive manufacturing has been of great interest in recent years since it offers the possibility to fabricate bioactive glasses or implants with arbitrary geometrical complexity in a short time. The main additive manufacturing methods include selective laser printing, inkjet printing or inkjet plotting on bioactive glasses or bioceramics. For all these methods, it is of the utmost importance to effectively set up the process parameters and monitor the system properties (such as particle size, viscosity of fluids, solvents) to develop layers, scaffolds, or implants with the desired thickness, pore structure, and functionalized surface able to promote tissue regeneration [72].

## 3. Applications and Clinical Trials

### 3.1. Clinical Applications of 45S5 Bioglass^®^

The two main commercially available bioactive glasses based on the 45S5 Bioglass^®^ composition, namely the PerioGlas^®^ (NovaBone Products LLC_Alachua FL, USA) and BioGran^®^ (BIOMET 3i™), belong to the CaO–Na_2_O–SiO_2_–P_2_O_5_ system [80]. They only differ in the size distribution of particles: PerioGlass has a particle size ranging from 90 to 170 µm, while the size of BioGran^®^ particles is in the 300 to 355 µm range [81].

PerioGlas^®^ was the first NovaBone^®^ particulate material on sale in the USA in 1993, while it obtained the CE Mark in Europe in 1995. This product was firstly used to replace bone loss resulting due to periodontal disease. In 1996, the FDA cleared additional indications for its application in dentistry to improve the alveolar ridge in tooth extraction sites. In 1992, J. Wilson et al. in [82] described, for the first time, the promising use of 45S5 Bioglass particulate to restore periodontal defects in the Patus monkey: the results showed that 45S5 particulate would be easily manipulated and, due to its hemostatic and osteoproductive properties, would allow restoration of both alveolar bone and periodontal muscle. Many other related studies based on animals reported similar results [12,19]. This research, performed using bioglass in animals, has been of great importance in order to understand the biological mechanisms involved in bio-integration and to establish the kinetics of surface reactions involved in the formation of a bond between the bone and the layer of hydroxyl-carbonate apatite (HCA) on the bioactive glass. Subsequently, this knowledge has been transferred to human clinical trials [82,83]. In a period of more than 20 years of clinical history, numerous trials have demonstrated the efficiency of PerioGlas^®^ in the regeneration of human intrabony defects such as periodontal osseous defects, pulp capping, sinus obliteration, and dentinal hypersensitivity. Table 2 reports a summary of the main clinical studies performed in the field of dental and maxillofacial applications based on the use of 45S5 and its derivate types, performed from 1997 up to the present [84,85,86,87,88,89,90,91,92,93,94,95,96,97,98,99,100,101,102,103]. The papers [60,61,62,63,64] evaluated the effectiveness of PerioGlas^®^ used in the treatment of intrabony defects in comparison with autogenous bone or open flap debridement. The results from these investigations indicated that the bioactive glass led to a significant defect depth reduction and to an increase in density and volume; in particular, BG usually showed significantly greater improvements in terms of gingival recession and osseous defect filling in the bioactive sites compared to the sites treated with surgical debridement only. However, Nevins et al. [99] found that bioglass used for the treatment of intrabony defects present around teeth led to limited bone formation; no evidence of new root cementum or signs of periodontal regeneration were observed. The different efficacy of BG is probably due to the slight difference in the composition of the available brands and to the size of the particulate, as explained later by Sohrabi et al. [92]. Cardioli et al. [100] suggested that bioactive glass BioGran^®^ and autogenous bone combined in ratio of 4:1, typically used to enhance the maxillary sinus floor, yields mineralized tissue of sufficient quality and volume. Mengel et al. [101] showed and compared the results of a 12-month clinical and radiological study dealing with the use of a bioabsorbable membrane and bioactive glass in the treatment of intrabony defects in patients with aggressive periodontitis. In [86], Mengel et al. published results obtained using the same system with a follow-up of 5 years. Both these studies provided evidence that bioactive glass is effective in the regenerative healing of periodontal lesions, as shown by the significant improvements in clinical parameters. Another work [102] showed that the use of BG covered with an HCA layer in bone regeneration to treat cortical and human maxillary cystic bone defects is more efficient than unmodified bioactive glass. This is due to the fact that the HCA layer is able to promote bone tissue regeneration, osteoblast adhesion, and graft material resorption, due to an increase in the selective adsorption of attachment proteins and growth factors. The results of Sculean et al. [103] showed the enhanced ability of enamel matrix derivative (EMD) and BG in the healing of human intrabony defects in comparison to a treatment using the BG alone. In particular, the development of new cementum due to the combination of the periodontal ligament and enhanced mineralization around the BG particles was observed in the case of EMD with BG. Gatti et al. [87] investigated the clinical use of PerioGlas^®^ granules with a size in the range 90–710 µm. The granules were placed in dental extraction sites before the placement of a dental implant in order to investigate the ability of PerioGlas^®^ to induce new bone formation, thus giving early fixation to the implant itself. The results showed the successful biodegradation of the glass (due to the formation of calcium phosphate acting as a scaffold for the colonization of osteoblasts), thus leading to well-loaded and stable implants. Banerjee et al. [88] compared the use of 45S5 Bioglass (Sylc^®^ Denfotex Research Ltd., London, UK) and sodium bicarbonate air-polishing powders in prophylaxis treatment on mandibular teeth. The results indicated that the bioactive glass powder was effective at reducing dental sensitivity and removing dental strain in patients with poor oral hygiene. In [89], the authors reported an investigation on the use of collagen barrier membranes in a mixture with autogenous bone graft (group 1) or autogenous bone graft and bioactive glass (group 2) in the treatment of intrabony defects. The results, based on the investigated parameters (probing depth reduction, clinical attachment level (CAL) gain, and defect resolution), were similar in both groups and suggested that significant improvements were observed after six months in all patients. This means that the BG can be mixed with autogenous bone when the amount of harvested bone is not sufficient for the treatment. The efficiency of bioactive glass PerioGlas in the treatment of human osseous defects was demonstrated in the clinical study performed by Subbaiah et al. [90]. In particular, the comparison of both treatments, the one with BG and that with open flap debridement, suggested that they have a similar ability to reduce the pocket probing depth. In addition, the bioactive glass showed the ability to improve the bone fill when compared to open flap debridement. The study by Stavropoulos et al. [91] dealt with the successful use of a BG and autogenous bone (in a 1:1 ratio) composite in implantation for transalveolar sinus augmentation. Some works [92,93] analyzed several clinical trials performed using a bioactive glass in the treatment of periodontal defects. A comparison with open flap debridement suggested that 45S5 used as a graft material leads to a significant improvement in both probing depth and clinical attachment level. Pereira et al. [94,95] compared the use of BioGran^®^ combined with an autogenous bone graft in a 1:1 ratio and an autogenous bone graft alone to increase/repair human maxillary sinus bone. In particular, the work focused on the cellular behavior by immunohistochemical assessment for vascular and osteoblastic activity developing during bone repair. The results suggested that both BioGran^®^ and its combination with the autogeneous bone graft in a 1:1 mixture are suitable as bone substitutes. The good results presented in this section [84,85,86,87,88,89,90,91,92,93,94,95,96,97,98,99,100,101,102,103] suggest that both PerioGlas^®^ and BioGran^®^ are able to enhance periodontal wound healing in intrabony defects, thus promoting the formation of new cementum associated with periodontal regeneration, without any adverse reactions to the product in the patient. Another product based on 45S5 Bioglass^®^ is NovaMin^®^, which was originally designed to inhibit the development of dental caries and induce remineralization of the tooth surface, reducing the cause of dentine hypersensitivity. In 2011, Glaxo-Smith-Kline launched a bioactive toothpaste called Sensodyne Repair and Protect based on the NovaMin technology: this product has shown superb efficiency in preventing dentinal pain and inhibiting gingivitis [104,105].

### 3.2. Clinical Applications of S53P4 Bioactive Glass or BonAlive^®^

Bioactive glass S53P4 is a bone-bonding, osteoconductive, and osteostimulative bone substitute with antibacterial properties. In this section, the main clinical applications of S53P4 bioactive glass (whose composition is reported in Table 1) in bone healing, vascularization, cartilage repair, and osteomyelitis treatment are presented and discussed. As previously explained, the activity of S53P4, analogously to the other bioactive glasses, is mainly based on surface reactions that occur after implantation and lead to the deposition of a calcium phosphate layer; this subsequently crystallizes into hydroxyapatite, which in turn is able to activate the osteoblasts and the formation of new bone. In particular, S53P4 is able to inhibit the bacterial growth, due to the dissolution process at the surface, able to release (alkali) ions, leading to an increase in pH and osmotic pressure [67]. Table 3 summarizes the most important clinical studies regarding this bioglass published from 2000 until now [40,52,107,108,109,110,111,112,113,114,115,116,117,118,119,120,121,122,123,124,125,126,127,128,129,130,131,132,133,134]. The analysis of the literature results based on clinical examinations such radiographs, computed tomography (CT), and magnetic resonance imaging (MRI) images shows that S53P4 may be successful in all the applications cited/reported in Table 3. As mentioned before, this material is mostly used in granules with a size in the range of 0.8 to 3.15 mm: the granules of the smallest size are primarily used in both hand and cranio-maxillofacial surgery, while the medium-sized and larger granules are primarily used in orthopedic, trauma, and chronic osteomyelitis surgery. However, the literature reports some use of S53P4 as nonporous plates or discs of various shapes, such as in [116,117,118,128], where plates are employed as repair materials for orbital floor fractures. In particular, Kinnunen et al. [117] used round and heart- or kidney-shaped implants (1 mm thick) in 28 patients. Aitasalo et al. [128] used implants of different sizes (20, 25, and 30 mm in diameter) having a thickness of 1 and 1.5 mm. The glass implants showed effectiveness similar to the traditional procedure based on cartilage harvested from the patient’s ear. Plates were also used for orbital floor or wall reconstruction in 49 patients (34 men and 15 women) [132]. The results obtained from the 2-year follow-up suggested that S53P4 could be considered a promising material for orbital reconstruction since it is slowly biodegradable, bioactive, and biocompatible. In addition, it prevents the growth of bacteria. Stoor et al. in [116] describe the successful use of S53P4 implants having a drop shape combined with post-surgery antibiotic therapy to reconstruct the orbital floor and recover the original volume of the orbit itself. The 32-month follow-up showed that the implants could retain the correct position without causing any adverse reaction or resorption associated with the use of this biomaterial. Despite these successes, commercial products are mainly in the form of granules rather than monolithic shapes (see Table 3).

Several clinical trials have compared the use of standard autografts with bioglass granules to understand the efficiency of the material in bone regeneration or reconstruction [107,109,134]. Clinical tests have studied the feasibility of S53P4 to be used as an obliteration material in chronic frontal sinusitis, which could not be treated otherwise [130,131]. In [130], Peltola and coworkers performed trials with BG granules, with sizes from 0.5 to 0.8 and from 0.8 to 1.0 mm, soaked with saline solutions. The promising results obtained suggested the use of the glass to reconstruct perforated chronically infected nasal septum, even though a need for more research regarding the antibacterial properties of the material emerged. In [129], Turunen et al. performed clinical trials on 17 patients using a 1:1 mixture of glass granules with a diameter in the range of 0.8 to 1 mm with autologous bone (AB) chips in previously damaged jawbone. The results showed that the recovery was faster with respect to the application of an autograft alone; the use of this mixture could be useful for the sinus floor augmentation procedure, since it allows a decrease in the amount of bone needed. Lindfors et al. [134] conducted a prospective randomized long-term follow-up of 14 years (surgeries made from 1993 to 1997) on 21 patients (11 in the BG group, 10 in the AB group) to evaluate the efficiency of S53P4 and autogenous bone used as bone graft substitutes in the treatment of a benign bone tumor. S53P4 granules, having a size in the range 1 to 2 mm, and then in the range of 1 to 4 mm (1–2, 2–3, or 3.15–4 mm), were used for the treatment of small bone tumors and larger tumors, respectively. The promising results from X-rays, MRI, and CT scans suggested that S53P4 did not alter bone growth in children, so that it can be considered a safe and well-tolerated bone substitute. Another comparison between S53P4 and autograft bone is given in [107], where nine patients with benign bone tumors in the hand were involved in a randomized and long-term study. No differences in terms of radiology were observed after 18 months from the surgery in the two groups. The materials did not cause any adverse effects during the follow-up of 14 years. In the BG group, CT suggested the formation of a thickened cortex: the bone marrow was mainly or partly fatty. In addition, an antibacterial effect was observed only in the BG group. In [108,109], a comparison between BonAlive^®^ and autograft efficiency for the treatment of depressed lateral tibia plateau fractures was considered. The glass granules (from 0.83 to 3.15 mm) were used to fill the bone defects. As reported in [109], some particles were still present after 1 year post-operation. The 11-year follow-up [108] showed that, in the BG group, healing had occurred, with similar bone regeneration compared to autograft: for this reason, BG granules can be considered a filler substitute material for autogenous bone in the treatment of lateral tibial plateau compression fractures. In [110,111], Frantzen et al. and Rantakokko et al. performed clinical trials in spondylodesis procedures for the treatment of spine burst fractures. Moreover, Kankare and Lindfors in [121] demonstrated that S53P4 could be a potential bone substitute in the treatment of severe spondylodiscitis. Frantzen et al. [110] used granules of this glass with a size from 1 to 2 mm for the treatment of severe spondylolisthesis (displacement of vertebrae). In particular, the work compared two groups: in the first, the BG was placed on the left side of the posterolateral fusion bed, while for the second group, autogenous bone (AB) was implanted on the right side. After 11 years of follow-up, the fusion rate for the bioactive glass was lower than for autograft (88% vs. 100%). In [111], the 10-year follow-up showed that almost half of the implants (5 out of 10) had reached complete fusion compared to all 10 autografts. Kankare et al. [121] report a work involving three patients with severe spondylodiscitis, whose vertebral defects were reconstructed using S53P4 and an expandable vertebral body replacement device. For two patients, only S53P4 was used to cover the expander, while for the third patient, a mixture of glass and autograft bone was used. The results, based on a relatively long follow-up, showed that all patients achieved a complete neurological recovery. These long-term results are of the utmost importance since they suggest that BG could be considered a good alternative as a bone graft extender in spinal surgery. As shown in several works [52,112,113,120,122,123,124], S53P4 is a suitable material to be applied in the treatment of mastoid cavity infections and cleaning problems in order to achieve a dry and safe ear. In particular, the use of this BG for obliteration surgery might be considered when a large cavity after canal-wall-down mastoidectomy should be avoided. Bernardeschi et al. [122] demonstrated that S53P4 is an effective, practical, and feasible biomaterial when used in primary and revision canal-wall-down and canal-wall-up mastoidectomies. In [123], S53P4 bioactive glass granules were used in cholesteatoma surgery in 67 patients. In line with Bernardeschi et al. [122], in a short-term evaluation, no evidence of undesirable effects on the inner ear were observed. In 96% of all cases, a safe and dry ear was achieved. Vos [122] investigated the use of S53P4 as a filler in the non-cholesteatomatous chronic otitis area during mastoid obliteration surgery. The good outcomes suggest that S53P4 mastoid obliteration led to satisfactory control of infections for a great number of patients as compared to patients that experienced only a mastoidectomy.

S53P4 was used as a bone graft substitute for its antibacterial properties in treating osteomyelitis, particularly when the bone quality of the vertebrae was reduced by bacterial infection (usually *Staphylococcus aureus*, *Mycobacterium tuberculosis*, *Candida tropicalis*) [40,52,114,115,119,121,133]. In spite of a mean follow-up of only 24 months (from 10 to 38 months), the primary results obtained by Lindfors et al. [133] show that this BG was well-tolerated: all the patients achieved good bone formation and only two suffered an infection caused by vascular problems in the muscle flap and by a deep hematoma, respectively. In [114], a short study demonstrated the success of S53P4 in concurrence with antibiotic therapy applied to treat three patients affected by chronic osteomyelitis. The results from the short follow-up of 14–21 months suggested that this glass could have significant potential in the treatment of osteomyelitis by virtue of its ability in the replacement of bone defects and potent antimicrobial properties. Romanò et al. [115] showed that patients treated only with S53P4 achieved infection eradication and less drainage, similar to those treated with calcium-based antibiotics.

Recently, Stoor et al. [125,126] achieved promising results with S53P4 granules in correcting bony lesions in the oral and maxillofacial region. In [125], Stoor described how to regenerate cystic bone cavities and bone defects in jaws, filling them with S53P4 granules. This work suggests that S53P4 can contribute to providing a good volume of the alveolar ridge and to improving the bone strength of the mandible. In addition, it shows that S53P4 could be used to support the teeth in the area close to the remodeled bone and to reduce the risk of infections and unpleasant reactions. Again, Stoor et al. [126] evaluated the use of S53P4 granules as a filling material in bilateral sagittal split osteotomies. The results, from the 4-year postoperative follow-up, showed effective bone regeneration and long-term stability of the osteotomy side and good remodeling of the inferior mandibular border. In particular, a stable occlusion in 88% of the patients and a good outcome in terms of aesthetics in 96% of the patients were observed.

### 3.3. Clinical Applications of Borate-Based Glasses (19-93B3 Bioactive Glass)

The composition of borate glass is reported in Table 1 [46]. Borate glasses are very interesting with respect to the technological viewpoint since they show a glass transition temperature (Tg) lower than silicate-based bioactive materials; hence, they can be sintered more easily than SiO_2_-based ones. The addition of specific oxides can be used to tailor the properties of these products [60,135]. Abdelghany et al. [136] investigated the corrosion behavior and the bioactivity of some borate glasses soaked in aqueous dilute phosphate buffer for different times considering the glass weight loss after immersion. FTIR was used to evaluate the conversion of the considered borate glasses into hydroxyapatite after the immersion. Richard [137] was the first author who discussed the replacement of silica with B_2_O_3_ in 45S5 glass. Fu et al. [138] demonstrated that the complete or partial replacement of silica by B_2_O_3_ leads to systems that are more reactive than 45S5 Bioglass^®^ and this can promote the faster growth of bone in in vivo tests performed in aqueous media; borate glasses can convert completely in hydroxyapatite in a few days. Moreover, these materials can stimulate angiogenesis, which favors bone healing [136]. Tailoring the composition of boron-based glasses, it is possible to obtain systems that control the release of minor elements, such as Zn, Ag, Cu, F, Mn, Sr, and B, able to favor bone growth or angiogenesis and to enhance the antibacterial properties [14,45,139] at a biologically suitable rate. A concern associated with borate bioactive glasses is the toxicity due to the high levels of (BO_3_)^3−^ released into the surrounding medium. Some studies report that in vitro biocompatibility tests show that borate glasses can be cytotoxic if the tests are carried out in static conditions. On the other hand, if the boron concentration is lower than 0.65 mM and the toxicity tests are performed in dynamic conditions, no cytotoxic effect is observed since borate ions are satisfactory diluted [140]. Ospina et al. [141] investigated the bioactivity and hence the dissolution behavior of a boron-based bioglass in SBF, tris solution, and in acidic media both under static and dynamic conditions. Furthermore, the increase in the dissolution rate corresponded to an increase in B content. The results confirm that the glass can degrade faster in dynamic conditions than in static ones. Although borate bioactive glasses have shown both mechanical and surface characteristic properties favorable for potential application in the biomedical area, most of the literature reports only results of in vivo or pre-clinical studies [42,45,142,143,144,145]. This is due to the fact that the area of bioactive borate glass science is still young and not sufficiently developed. Recently, MIRRAGEN (DermaFuse, now known as Mirragen), a borate-based glass microfiber wound dressing (MIRRAGEN, ETS Wound Care LLC, U.S.), has been approved by the Food and Drug Administration (U.S.) for the treatment of acute and chronic wounds such as diabetic ulcers [4,5,44]. The borate material dissolves upon exposure to body fluid, releases ions at the wound area, and is then absorbed by the surrounding tissue. In particular, borate glasses are used as scaffolds for tissue engineering, providing the required support and being able to promote the growth of new tissues through angiogenesis. This kind of bioactive glass may be also an interesting carrier for antibiotic delivery [28,61,72]. Some studies report [44,146,147,148,149] very encouraging clinical results and conclude that MIRRAGEN is effective in the healing of wounds that could not be recovered by conventional treatment. Although promising results have been achieved, such as bone growth and the successful healing of chronic wounds, further research is required to better understand the borate-based glass mechanism in wound healing and the cytotoxicity of these systems.

## 4. Conclusions

This review provides a literature overview of different applications of some of the most common bioactive glass compositions (in particular, 45S5, S53P4, and borate-based glass 19-93B3), and their clinical outcomes/results in the treatment of different human pathologies. The main focus is on their use in dentistry, reconstructive surgery (bone regeneration), and the treatment of infections. Section 3.1 deals with the outcomes of 45S5 bioactive glass or Bioglass^®^/Biogran^®^ used in clinical trials for the treatment of bone or dental trauma and diseases such as osteoporosis, cancer, and infections to replace damaged tissues: in this context, the results show that this bioactive glass can be successfully employed as a substitute or in combination with autologous bone. Section 3.2 provides evidence that S53P4 bioactive glass, based on the long-term follow-up studies reported in randomized controlled trials, is safe and well-tolerated in the treatment of bone defects in craniofacial surgery, osteomyelitis, and the grafting of bone tumor defects. In the last section, it has been shown that the fibrous borate bioactive glass is effective in the healing of long-term venous stasis/acute ulcers.

To conclude, 45S5, S53P4, and borate-based glasses 19-93B3 are versatile replacement materials in the treatment of human defects since they are available in different forms and shapes and able to satisfy the needs of users. These bioactive glasses represent an exciting and evolving field of study. Current and potential future applications depend on the specific compositions of the given biomaterial, so that studies of the utmost importance have been performed to understand the interactions between glasses and the host cells; the importance of dopant ion diffusion on the bioactivity of such glasses is under investigation, too. Other innovative glass compositions have been proposed in recent years, also doped with the so-called therapeutic ions: these systems will be the focus of present and future clinical trials. In particular, it will be very important to assess the effect of some specific dissolution products from bioactive glasses on the in vivo outcomes. Finally, it is worth noting that the optimization of the glass composition should include the analysis of potential residual stresses due to the mismatch of coefficients of thermal expansion [150,151] in composites and/or in support/coating systems (such as BG-coated prostheses [152,153]).

## Figures and Tables

**Table 2:** The main clinical studies, performed in the field of dental and maxillofacial applications, based on the use of 45S5 and its derivate types (from 1997 to present).

Refs.	Glass Composition	Material and Treatment	How/Where and How Long Was the Biomaterial Implanted?	Conclusions	Notes
[84]	45S5	- BG particle size between 90 and 710 µm; - Following flap reflection, root planing, and removal of chronic inflammatory tissue; - In one group of patients, the test defects were restored with the bioactive glass; - Replacement and suture of mucoperiosteal flaps; - Use of a periodontal dressing.	- 20 patients between 23 and 55 years old with periodontal intrabony defects (44 sites); - 2 groups: only in 1 group, defects were restored with the bioactive glass; - Follow-up: weekly and at 3 months, 6 months, 9 months, and 1 year post-surgery.	- Significant increase in radiographic density and volume between the defects treated with Perioglas^®^; - Significant improvements in probing pocket depth (PPD) and probing attachment level (PAL) both in experimental and control sites; - Efficiency of bioactive glass used as an adjunct to conventional surgery in the treatment of intrabony defects.	
[85]	45S5	Bioglass particulates (particle size between 90 and 710 μm); Periodontal osseous defects.	12 patients; Follow-up: initially at 3, 6, and 24 months post-treatment.	- 3.33 mm reduction in mean probing depth; - Mean attachment gain of 1.92 mm; - Mean radiographic bone fill of 3.47 mm; - Stable results over the 24-month period.	Ease handling of BG during treatment and excellent tissue response.
[86]	45S5	Bioglass and polymeric membranes (Millipore^®^ filter or Gore-tex^®^ membrane)	- Patients with serious periodontitis; - Defects at molars and incisors not considered; - Age: from 32 to 62 years old; - Some defects were treated with the polymeric membranes and some with BG alone; - Follow-up at 6 months and every year for 5 years after surgery.	- A reduction in probing depth; - A gain at clinical attachment level (CAL); - The periodontal intrabony defects were found to be filled significantly more in the BG group.	
[87]	PerioGlas^®^	Granules, with particle size from 90 to 710 µm, employed as fillers after tooth extraction.	- 3 patients; - Follow-up: biopsies of the bone in the site of tooth extraction treated with glass granules after 6 months.	- Good bioactivity of PerioGlas^®^ granules confirmed by new bone formation and glass biodegradation. - Follow-up after two years: successful load of the implants; evidence for implant stability.	
[88]	45S5	- Calcium sodium phosphosilicate powder, (CaNaO6P-Si) was compared with bioglass in powder; - Air-polishing treatment, i.e., a non-invasive technique used to remove staining and harmful plaque from the teeth—a mixture of compressed air, water, and fine powder is used; - Mandibular teeth prophylaxis treatment to investigate the occlusion of open dentine tubules; - Maxillary teeth with untreated sites as reference area to occlude open dentine tubules, whilst leaving calcium phosphate ions adhered to the surface in a single application via an air-polishing treatment.	- 25 patients (age 18–64 years old) divided into two groups: the first with good oral hygiene and the second with poor oral hygiene; - Follow up: before and after the treatment and a 10-day recall.	Bioactive glass air-polishing was more clinically and statistically effective in desensitizing; removal of stain with a significant reduction in dental sensitivity in the poor oral hygiene patient subgroup.	45S5 provided patient comfort during the treatment.
[89]	PerioGlas^®^	Treatment of moderate to severe chronic periodontitis.	- 22 patients (12 males and 10 females from 20 to 49 years); - Thirty-two periodontal intrabony defects; - Follow up: after 6 months.	- Significant probing depth reduction, clinical attachment level gain (CAL), and defect resolution in all groups, with better outcomes in the group with autogenous graft (pure); - Similar clinical attachment gain for the two tested groups.	Mixture of autogenous bone and bioactive glass effectiveness if the amount of the harvested bone is not sufficient.
[90]	PerioGlas^®^	Bioactive alloplast; Treatment of periodontal osseous defects.	- 8 volunteers (20–65 years old) with at least two periodontal osseous defects; - Follow-up: pre- and postoperatively after 3, 6, and 9 months; - Radiographs of each defect, measure of defect depth from the alveolar crest to the base of the bone defects using a Williams graduated periodontal stent.	- Comparison between the sites treated with bioactive glass and the ones treated only with open flap debridement: no significant differences between the two methods; - Significant improvement in bone fill when the bioactive glass is used; - Bioactive glass was well-tolerated by the gingival tissues.	
[91]	BioGran^®^ (particles with particle size between 300 and 355 µm) and autogeneous bone composite.	- 1:1 ratio composite of bioglass and autogenous bone; - Implantation for transalveolar sinus augmentation.	31 patients	The tissue fractions occupied by newly formed bone (mineralized tissue bone marrow), soft connective tissue, residual biomaterial empty spaces, and debris inside the sinus cavity or the transalveolar osteotomy were estimated.	No collateral effects on bone formation due to sinus augmentation with a bioglass and autogenous bone composite; similar density as that reported for other commonly used bone substitutes.
[92]	45S5	- Surgical treatment of periodontal intrabony defects. - Bioglass particulates; - Periodontal osseous defects: treatment of intrabony and/or furcation defects; - A review on a collection of 15 clinical trials.	- Treatment of intrabony and/or furcation defects; - Follow-up: initially at 3, 6, and 12 months post-treatment.	Measures of probing depth (PD) and clinical attachment levels.	BG imparts a significant improvement in both probing depth and/or clinical attachment level measures with respect to open flap debridement treatment.
[93]	PerioGlas^®^	- Evaluate the efficacy of PerioGlas^®^ and compare it to open debridement as control; - Treatment of human periodontal osseous (three and two wall) defects in South Indian population.	- 10 patients (30–45 years old) exhibiting vertical osseous defects; - 10 sites (experimental) received PerioGlas^®^ material after open flap debridement and 10 sites with open flap debridement (controls); - Follow-up: from 6 weeks to 9 months postoperatively.	- Statistically significant improvement in both clinical and radiographic parameters for both groups; - Experimental sites showed better results when compared with control.	
[94]	BioGran^®^	- Three different materials were employed: Biogran alone, a 1:1 combination of Biogran and autogenous bone graft, and autogenous bone graft alone; - Scope: augmenting maxillary sinus height.	30 patients: Group 1: 10 maxillary sinuses grafted with Biogran. Group 2: 10 maxillary sinus grafted with Biogran added to autogenous bone graft in a 1:1 ratio. Group 3: 10 maxillary sinus grafted with autogenous bone graft alone. Biopsy samples were collected at the time of dental implant placement and after 6 months.	- New bone formation in the pristine bone region, in the intermediate region, and in apical region; - Connective tissue well-cellularized in all regions with more marrow areas in the apical; - Presence of a periphery line of osteoblastic cells; - Similar outcomes: no statistically significant difference in the new bone formed among the groups or among the 3 regions within each group.	- Biogran^®^ and its combination with autogenous bone graft 1:1 are good bone substitutes due to their similarity to autogenous bone graft; - Both investigated grafts lead to a good placement of dental implants.
[95]	BioGran^®^	- Bioactive glass with autogenous bone graft in a 1:1 ratio; - Autogenous bone block grafts harvested under local anesthesia; - The maxillary sinus bone augmentation was performed in accordance with the surgical procedure of Boyne and James [106]; - Therapy with antibiotics.	- 29 patients (35 maxillary sinuses); - 12 maxillary sinuses were grafted with bioactive glass (group 1), 9 with bioactive glass mixed with autogenous bone graft 1:1 (group 2), and 12 with autogenous bone graft (group 3); - Follow up: 15 days and 6 months after the procedure.	- Similar outcomes for maxillary sinus bone augmentation in all groups. However, the addition of 50% bioactive glass to autogenous bone graft improved the microarchitecture of the graft; - The mixture BioGran^®^/autogenous bone graft decreased the resorption volume and improved the trabecular bone microarchitecture.	Further studies are necessary to demonstrate the cellular activity and the osteogenic potential of these biomaterials.
[96]	45S5	- BG compared to demineralized freeze-dried bone allograft (DFDBA); - Moderate to chronic intrabony periodontal defects (periodontitis).	15 patients (6 males and 9 females, aged 30 to 63 years).	Results in 6 months similar to those of DFDBA.	
[97]	45S5	- Test BG implanted sites vs. unimplanted sites; - Periodontal osseous defects.	- 95 defects in 16 healthy adults; - Radiographs and soft tissue presurgical measures repeated at 6, 9, and 12 months.	- Significantly greater defect fill and defect depth reduction with BG compared to the control sites; - Significant improvement in clinical parameters with BG compared to open flap debridement.	
[98]	BioGran^®^	- Treatment for sinus augmentation.	- 10 patients underwent bilateral grafting; - A 1:1 mixture of autogenous bone particles (from iliac crest) and bioglass particles at one side (experimental side) were used; - Only bone particles at the other side (control side, split mouth design); - Follow-up: 4, 5, 6, and 16 months.	Mixture seems a promising alternative to autogenous bone only when low amounts of bone tissue are available for sinus augmentation.	Bioactive glass particles can help to achieve sufficient bone quantity and quality, provided that a healing time of at least 6 months is used before the implant placement.
[99]	PerioGlas^®^	- Presurgical phase of scaling and root planing therapy; - Intrabony defects around 5 teeth.		- Encouraging clinical results only in one case, where the intrabony region demonstrated new cementum formation and new connective tissue attachment; - Minimal new bone formation; - Histologic analysis: bioactive glass ceramic seems to have limited regenerative properties as periodontal grafting material.	
[100]	BioGran^®^ combined with autogenous bone.	- Bioactive glass and autogenous bone in a 4:1 ratio; - Composite graft of approximately 70–80% Biogran^®^ and approximately 20–30% of particulate autogenous bone mixed with blood coagulum was then introduced and carefully packed without excessive pressure into the posterior part of the sinus cavity and into the anterior part; - Post-surgery antibiotic therapy.	- 12 patients; - 3–5 mm of alveolar crestal bone height in the posterior maxilla prior to grafting; - A total of 27 implants; - 26 implants were stable; - 12 months of follow-up.	- None of the patients had postoperative complications besides normal swelling and inflammation at the surgical sites; - Sufficient quality and volume of mineralized tissue; - One implant failed during the prosthetic phase; - Combination bioactive glass and bone used in one-stage sinus augmentation yields sufficient quality and volume of mineralized tissue.	
[101]	45S5	Generalized aggressive periodontitis.	- 12 patients (9 females, 3 males); - 30 defects: 15 treated with the polymeric membrane (RXT group) and 15 with the bioactive glass (PG group); - Follow-up: 6 and 12 months after surgery.	- Highly significant improvements in the parameters: probing depth; clinical attachment level; filling of the intrabony defects by radiographs from alveolar crest (xCA) to the defect base (xBD) were taken; - Radiography: the defects were found to be filled significantly more in the BG group.	Both the bioabsorbable membrane and bioactive glass are suitable for the treatment of periodontal intrabony defects
[102]	- 45S5 particles covered with a layer of amorphous calcium phosphate (BG-ACP); - 45S5 particles covered with a layer of hydroxycarbonate apatite (BG-HCA); - unmodified bioactive glass (BG) particles (as a control).	- BG glass was prepared by means of a classical melting route; - BG glass was ground and sieved to a final particle size between 150 and 300 μm; - Treatment of intrabony radicular cyst in the anterior maxilla; - Surface chemistry of the bioactive glass was modified by immersion in a simulated body fluid solution (SBF) for different periods of time.	- 30 patients divided into 3 groups of 10 patients (6 men and 4 women in each group); - Follow-up examinations from 1 day to 24 weeks post-surgery.	- Both clinical and animal studies were performed; - Improvement in biologic performance of bioactive glass covered with an HCA layer; - The modified BG accelerated bone formation and graft material resorption better than unmodified bioactive glass; - Bone regeneration and graft resorption in cortical bone defects and maxillary cystic cavities were significantly greater in defects grafted with BG-HCA than in defects grafted with BG-ACP or unmodified BG.	More rapid bone regeneration observed associated with BG-HCA, which was probably due to the ability of the HCA layer to enhance the selective adsorption and attachment of proteins and growth factors; this fact seemed to stimulate osteoblast adhesion and the subsequent bone deposition.
[103]	Mixture of enamel matrix derivative (EMD) and 45S5 bioactive glass (BG) or BG alone	Intrabony defects around teeth.	6 patients: 5 persons with one- and two-walled (five patients) intrabony defects and 1 patient with three-walled intrabony defects	- Samples treated with BG: epithelial down-growth and good connective tissue encapsulation of the graft material for 3 patients; formation of cementum and periodontal ligament (1 patient); formation of bone-like tissue around the graft particles; - No evidence for direct contact between BG particles and root surface; - BG alone has low potential to facilitate periodontal regeneration; - EMD + BG: significant cementum formation due to periodontal ligament; good remineralization around the BG particles.	

**Table 3:** The main clinical studies based on the use of S53P4 and its derivate types from 2000 to present.

Refs.	Material and Treatment	How/Where and How Long Was the Biomaterial Implanted?	Conclusions	Notes
[40]	S53P4 granules in the range from 0.8 to 3.15 mm. S53P4 nonporous plates or discs of different shapes.	S53P4 as bone graft substitute for osteomyelitis treatment.	Very good outcomes in different applications of glass.	
[52]	S53P4 granules.	- 27 patients (18 males, 9 females) with diagnosed osteomyelitis of the long bones; - Follow-up: from 15 days to 24 months.	Absence of local or systemic side effects connected with the use of the bioactive glass.	
[107]	S53P4 granules vs. autograft bone. Treatment of benign hand bone tumors.	- 9 patients; - Follow-up of 14 years.	- No material-related adverse effects; - S53P4 is safe and well-tolerated; - Good results in long term.	
[108]	S53P4 and autograft bone (AB) as bone graft substitutes in depressed tibial plateau fractures.	- All patients had sustained tibial plateau fractures with a joint-line depression of >3 mm; - Fifteen patients (5 patients in the S53P4 group, 10 patients in the AB group); - 11-year follow-up study.	- No significant difference in the tibial–femoral angle or deviation of mechanical axes between the two groups; - S53P4 eligible as a bone substitute in depressed lateral tibial plateau fractures with good functional and radiological long-term results.	
[109]	S53P4 produced by melting (granules with size in the range from 0.83 to 3.15 mm) used as filler in the treatment of the lateral tibial plateau compression fractures.	- 14 cases (7 females, 7 males, aged from 25 to 82 years) treated with bioglass fillers or bone (the control group); - Follow up: 12 months, with radiological analysis pre- and postoperatively, at 3 and 12 months.	Bioactive glass granules revealed good properties as filler material.	
[110]	S53P4 and autogenous bone (AB) for posterolateral spondylodesis treatment.	- 17 patients (12 women, 5 men); - Follow-up: 11 years (from 1996 to 1998).	A solid bony fusion was seen on tomography scans on the AB side in all patients and on the BG side in 12 patients. Glass used as a bone graft extender was demonstrated to be a good alternative in spinal surgery.	
[111]	S53P4 and autogenous bone (AB) as control. Bioactive glass implant on the left side of the fusion bed and AB implant on the right side. Treatment of fractures in the unstable lumbar spine burst.	- 10 patients (9 males, 1 female); - Follow-up: from 1996 to 1998.	Total fusion rate of 71% of all fused segments in the BG group.	
[112]	Glass granules moistened with saline used in mastoid obliteration surgery.	- 26 people (12 male, 14 female), median age 50; - 25 patients with chronic otitis media and 1 patient with cerebrospinal fluid leakage without chronic infection; - 20 patients had previous surgery; - Median follow-up: 55 months (range from 1 to 182 months).	96% of patients: dry, safe ear and only intermittent otorrhea.	
[113]	S53P4 in granules. Obliteration of mastoidectomy cavity.	- 16 patients (6 males and 9 females, adults). - Mean follow-up time: 2.2 years.	Ears were dry within a month after the surgery.	
[114]	S53P4 in granules	- 3 patients (2 males and 1 female) with chronic osteomyelitis. - Follow-up: 14–21 months.	Good integration of the bioglass with the bone.	Antibiotic post-operation therapy for the treatment of chronic osteomyelitis.
[115]	S53P4 in granules to fill bone defects after debridement in bone infections.	27 patients affected by chronic osteomyelitis.	For treatment with bioactive glass without local antibiotics: similar achievement in terms of infection eradication and less drainage than treatment with 2 different antibiotic-loaded calcium-based bone substitutes.	No antibiotic to treat bone defects in infections.
[116]	Implants of bulk S53P4 were made in 2 sizes with a thickness of 1 mm and rounded edges.	- 20 patients with a fracture of orbital floor (isolated blow-out fracture of the orbital floor or with a combined zygomatico-orbitomaxillary complex fracture). - Follow-up of 32 months.	- No complications related to the implant. - None of the patients had persisting diplopia. - Very good clinical results.	Antibiotic therapy.
[117]	S53P4 or autogenous cartilage implants. Bioactive glass plates 1 mm thick, round-, heart-, or kidney-shaped. Orbital floor fractures with persistent diplopia, enophthalmos, and/or infraorbital nerve paresthesia.	- 28 patients treated from 1991 to 1995; - 3 cases of persistent diplopia, 2 of infraorbital nerve paresthesia, and 1 of enophthalmos.	- Stable orbital and maxillary sinus volume; - Bioactive glass implants are well-tolerated and S53P4 seems to be a promising repair material for orbital floor fractures; - Favorable healing and new bone formation.	Cranio-maxillofacial surgery required proper closure to avoid exfoliation of material. Bioactive glass demonstrated to be a well-tolerated and promising material for orbital floor fractures.
[118]	A continuous glass fiber-reinforced composite (FRC) that contained S53P4 as one component. Bioactive glass granules: 500–800 μm (BonAlive^®^). Resin matrix of FRC: a highly polymerized cross-linked dimethacrylate biocompatible polymer.	- 7 patients underwent reconstruction of cranial defects in children (between 2010 and 2013); - Follow-up: from 1 week to 12 months.	FRC implant containing particles of glass: safe and a functional option for reconstruction of large skull bone defects.	The implant was moistened in cloxacillin solution. After operation: treatment with intravenous cloxacillin for 3 days.
[119]	S53P4 granules (BonAlive^®^).	- The aim of the work was to evaluate the behavior of the glass as bone substitute for the treatment of infection in humans; - 41 patients were treated between 2007 and 2013; - Follow-up of 21 months.	Three cases of osteomyelitis recurred. In two cases, a new procedure was performed. No complication directly related to the bioactive glass was reported.	Mean volume of particles inserted: 16.8 mL.
[120]	S53P4 granules (BonAlive^®^) Treatment of cavity obliteration.	- 133 cases; - Follow-up: from 4 months to 33 months.	A combination of S53P4/cartilage led to efficiency for cavity obliteration.	
[121]	BonAlive^®^ granules in mixture with autologous bone (healthy pieces of the laminectomy bone were used). Treatment of posterolateral spondylodesis and unstable lumbar fractures.	- 3 patients; - Glass with different particle sizes (from 0.8 to 3.15 mm) was used to prepare several glass/bone mixtures; - Follow-up: 4 years for patient 1, 1 year and 8 months for patient 2, and 2 years and 2 months for patient 3.	Adequate fusion both when glass granules were used alone and in mixture with autologous bone. Fusion rate of 88% when S53P4 was used as a stand-alone bone substitute and in the treatment of posterolateral spondylodesis; fusion rate of 71% for treatment of lumbar fractures.	Follow-up of patient 2 interrupted by his death.
[122]	- Granules of glass (0.5–0.8 mm in diameter); - Removal of the lesions (cholesteatoma and/or inflammatory mucosa); - Removal of all visible pathologic mucosa in the cavities; - Reconstruction of the middle ear (tympanic drum + ossiculoplasty when needed); - Cartilage and fibrous tissue coverage of bioglass granules in contact with the skin of the external auditory canal.	- Patients treated for mastoid and epitympanic obliteration; - Forty-one cases (39 patients) operated between May 2013 and January 2015; - Follow-up: 1 year.	At 1 year, all patients presented a well-healed external auditory canal, with an intact tympanic membrane.	5-year follow-up is necessary to evaluate the long-term results of the obliteration.
[123]	S53P4 granules (BonAlive^®^); Fibrin glue used to cover the S53P4 granules.	- 67 patients: 18 young (age <17 years) and 49 adult (age ≥17 years) patients treated for cholesteatoma underwent tympanomastoidectomy with mastoid obliteration in the period 2012–2015 at the Diakonessenhuis, Utrecht, the Netherlands; - Follow-up period: 22 months; range from 12 to 54 months.	- Absence of pre- and postoperative complications; - Cholesteatoma recidivism was observed in 6% of the young patients (four ears); - Dry ear was achieved in 96% of all cases.	Pre- and postoperative antibiotic therapy. -In line with Bernardeschi et al. [122], the authors did not observe any signs of adverse effects on the inner ear.
[124]	S53P4 as filler material in mastoid obliteration surgery to treat non-cholesteatomatous chronic otitis media.	- 94 patients (96 ears): 23 people (23 ears) treated with S53P4; 71 patients (73 ears) as controls; - Surgery between 2005 and 2015.	- Significant improvement in the achievement of cured ear as compared to mastoidectomy alone; - No adverse events when the glass was used in obliteration.	
[125]	S53P4 bioactive glass granules from 500 to 800 µm or 1.0 to 2.0 mm in size used as cavity filler of bony defects. The granules of the BG S53P4 were moistened with the venous blood of patient or saline before filling the cavity.	- 20 patients (21 cases); - Glass granules with different sizes were used (from 0.5 to 2 mm).	S53P4 provides effectiveness in the treatment of large bone defects and leads to infection-free and reliable bone regeneration.	Postoperative antibiotic prophylaxis. The material is easier to handle with a smaller granule size.
[126]	S53P4 granules moistened with saline solution. Stabilization of the S53P4 granules with a fibrin sealant made of human fibrinogen and human thrombin. Tissue glue to prevent migration of the granules, resorbable suture to close the wound.	- 25 patients with class II dentoskeletal deformities; - Up to 4 years of postoperative follow-up.	- Good healing, bone regeneration, and stability of the osteotomy sites; - Recontouring of the inferior mandibular border; - Stable occlusion in 88% of the patients; - Good aesthetic outcome of the osteotomy sites in 96% of the cases.	Postoperative antibiotic prophylaxis.
[127]	S53P4 as filling agent.	- 18 children with primary aneurysmal bone cyst surgery between 2008 and 2013; - Follow-up of 2 years.	- No intraoperative or postoperative complications due to the implanted material; - Bone growth was not affected by the use of bioactive glass.	
[130]	S53P4 granules moistened with saline. Bioactive glass used as obliteration material.	- 42 patients (20 women and 22 men) affected by chronic frontal sinusitis, not curable with other means of treatment; - Bicoronal (35 patients) or eyebrow (7 patients) incision; - Follow up: from 3 months to 12 years.	- Promising results. In particular, the microscope analysis revealed new bone formation; - S53P4 seems to be a reliable material for total bony sinus obliteration; - Need for more research on the antibacterial property of bioactive glass.	Postoperative antibiotic therapy (5 days).
[131]	S53P4 granules. Treatment of frontal sinus.	Follow-up of 13 years.	- Formation of new bone between biomaterial granules. - No foreign body reaction.	
[132]	S53P4 plates. Reconstructive surgery of the orbit.	- 49 patients (34 men and 15 women); - Reconstruction of orbital floor or wall; - Follow-up from 1 week to 24 months after the operation.	- No sign of microbial growth; - Bioglass plate well-tolerated and suitable as reconstruction material.	Drawback: plates are brittle and rigid, and difficult to be shaped by a surgeon.
[133]	S53P4 in treatment of osteomyelitis. Verified chronic osteomyelitis in the lower extremity and the spine.	- 11 patients; - Follow-up: 24 months.	- S53P4 is a good and well-tolerated bone substitute. - Good preliminary results.	
[134]	S53P4 granules (BonAlive^®^) and AB harvested from the iliac crest used as a filling material. Size of the glass granules was 1–2 mm in small bone tumors; glass granule sizes of 1–2, 2–3, or 3.15–4 mm in tumors of high extension.	25 patients (9 females, 16 male); among them, 14 were treated with bioglass.	S53P4 is a good material of choice in benign bone tumor surgery both in children and adults.	

## Data Availability

Not applicable.

## References

[1] Hoppe A., Boccaccini A.R. (2016). Chapter Bioactive Glasses as Carriers of Therapeutic Ions and the Biological Implications. Bioactive Glasses.

[2] Ali S., Farooq I., Iqbal K. (2014). A review of the effect of various ions on the properties and the clinical applications of novel bioactive glasses in medicine and dentistry. Saudi Dent. J..

[3] Zhao X., Courtney J., Qian H. (2011). Bioactive Materials in Medicine: Design and Applications.

[4] Boccaccini A.R., Blaker J. (2005). Bioactive composite materials for tissue engineering scaffolds. Expert Rev. Med. Devices.

[5] Miguez-Pacheco V., Hench L.L., Boccaccini A.R. (2015). Bioactive glasses beyond bone and teeth: Emerging applications in contact with soft tissues. Acta Biomater..

[6] Vollenweider M., Brunner T.J., Knecht S., Grass R.N., Zehnder M., Imfeld T., Stark W.J. (2007). Remineralization of human dentin using ultrafine bioactive glass particles. Acta Biomater..

[7] Profeta A.C., Prucher G.M. (2015). Bioactive-glass in periodontal surgery and implant dentistry. Dent. Mater. J..

[8] Jones J.R., Clare A.G. (2012). Bio-Glasses: An Introduction.

[9] Jones J.R., Brauer D.S., Hupa L., Greenspan D.C. (2016). Bioglass and Bioactive Glasses and Their Impact on Healthcare. Int. J. Appl. Glas. Sci..

[10] Hench L.L. (1998). Bioactive materials: The potential for tissue regeneration. J. Biomed. Mater. Res..

[11] Hench L.L. (2002). Third-Generation Biomedical Materials. Science.

[12] Jones J.R. (2015). Reprint of: Review of bioactive glass: From Hench to hybrids. Acta Biomater..

[13] Islam T., Felfel R.M., Neel E.A.A., Grant D., Ahmed I., Hossain K.M.Z. (2017). Bioactive calcium phosphate–based glasses and ceramics and their biomedical applications: A review. J. Tissue Eng..

[14] Hoppe A., Güldal N.S., Boccaccini A.R. (2011). A review of the biological response to ionic dissolution products from bioactive glasses and glass-ceramics. Biomaterials.

[15] Kaur G., Pandey O., Singh K., Homa D., Scott B., Pickrell G. (2014). A review of bioactive glasses: Their structure, properties, fabrication and apatite formation. J. Biomed. Mater. Res. Part A.

[16] Gerhardt L.-C., Boccaccini A.R. (2010). Bioactive Glass and Glass-Ceramic Scaffolds for Bone Tissue Engineering. Materials.

[17] Baino F., Novajra G., Miguez-Pacheco V., Boccaccini A.R., Vitale-Brovarone C. (2016). Bioactive glasses: Special applications outside the skeletal system. J. Non Cryst. Solids.

[18] Boccaccini D., Cannio M., Bernardo E., Boccaccini A.R. (2021). Glass and Glass-Ceramic Matrix Composites for Advanced Applications: Part II: Applications. Encyclopedia of Materials: Technical Ceramics and Glasses.

[19] Hench L.L. (2006). The story of Bioglass^®^. J. Mater. Sci. Mater. Med..

[20] Abbasi Z., Bahroloolum M.E., Shariat M.H., Bagheri R. (2015). Bioactive Glasses in Dentistry: A Review. J. Glas. Dent. A Rev..

[21] Gul H., Zahid S., Kaleem M. (2015). Bioglass, a New Trend Towards Clinical Bone Tissue Engineering. Pak. Oral Dent. J..

[22] Sergi R., Bellucci D., Cannillo V. (2020). A Comprehensive Review of Bioactive Glass Coatings: State of the Art, Challenges and Future Perspectives. Coatings.

[23] Sergi R., Bellucci D., Cannillo V. (2020). A Review of Bioactive Glass/Natural Polymer Composites: State of the Art. Materials.

[24] Wang Y., Zhao Q., Han N., Bai L., Li J., Che E., Hu L., Zhang Q., Jiang T., Wang S. (2015). Mesoporous silica nanoparticles in drug delivery and biomedical applications. Nanomed. Nanotechnol. Biol. Med..

[25] Hum J., Boccaccini A.R. (2012). Bioactive glasses as carriers for bioactive molecules and therapeutic drugs: A review. J. Mater. Sci. Mater. Electron..

[26] Ferrando A., Part J., Baeza J. (2017). Treatment of Cavitary Bone Defects in Chronic Osteomyelitis: Bioactive glass S53P4 vs. Calcium Sulphate Antibiotic Beads. J. Bone Jt. Infect..

[27] Li Y., Placek L.M., Coughlan A., Laffir F.R., Pradhan D., Mellott N.P., Wren A.W. (2015). Investigating the influence of Na+ and Sr2+ on the structure and solubility of SiO_2_–TiO_2_–CaO–Na_2_O/SrO bioactive glass. J. Mater. Sci. Mater. Electron..

[28] Jung S.B. (2012). Bioactive Borate Glasses. Bio-Glasses: An Introduction.

[29] Hupa L. (2011). Melt-derived bioactive glasses. Bioactive Glasses: Materials, Properties and Applications.

[30] Hench L.L. (2013). Chronology of Bioactive Glass Development and Clinical Applications. New J. Glas. Ceram..

[31] Hench L.L., Jones J.R. (2015). Bioactive Glasses: Frontiers and Challenges. Front. Bioeng. Biotechnol..

[32] Chen Q.Z., Thompson I.D., Boccaccini A.R. (2006). 45S5 Bioglass^®^-derived glass–ceramic scaffolds for bone tissue engineering. Biomaterials.

[33] Fabbri P., Cannillo V., Sola A., Dorigato A., Chiellini F. (2010). Highly porous polycaprolactone-45S5 Bioglass^®^ scaffolds for bone tissue engineering. Compos. Sci. Technol..

[34] Day R.M., Boccaccini A.R., Shurey S., Roether J.A., Forbes A., Hench L.L., Gabe S.M. (2004). Assessment of polyglycolic acid mesh and bioactive glass for soft-tissue engineering scaffolds. Biomaterials.

[35] Bellucci D., Sola A., Salvatori R., Anesi A., Chiarini L., Cannillo V. (2014). Sol–gel derived bioactive glasses with low tendency to crystallize: Synthesis, post-sintering bioactivity and possible application for the production of porous scaffolds. Mater. Sci. Eng. C.

[36] Bellucci D., Cannillo V. (2018). A novel bioactive glass containing strontium and magnesium with ultra-high crystallization temperature. Mater. Lett..

[37] Sergi R., Bellucci D., Salvatori R., Maisetta G., Batoni G., Cannillo V. (2019). Zinc containing bioactive glasses with ultra-high crystallization temperature, good biological performance and antibacterial effects. Mater. Sci. Eng. C.

[38] Bellucci D., Veronesi E., Dominici M., Cannillo V. (2020). A new bioactive glass with extremely high crystallization temperature and outstanding biological performance. Mater. Sci. Eng. C.

[39] Sergi R., Bellucci D., Salvatori R., Anesi A., Cannillo V. (2020). A Novel Bioactive Glass Containing Therapeutic Ions with Enhanced Biocompatibility. Materials.

[40] Van Gestel N., Geurts J., Hulsen D.J.W., van Rietbergen B., Hofmann S., Arts J.J. (2015). Clinical Applications of S53P4 Bioactive Glass in Bone Healing and Osteomyelitic Treatment: A Literature Review. BioMed Res. Int..

[41] Xynos I.D., Edgar A.J., Buttery L.D.K., Hench L.L., Polak J.M. (2001). Gene-expression profiling of human osteoblasts following treatment with the ionic products of Bioglass^®^ 45S5 dissolution. J. Biomed. Mater. Res..

[42] Zhao S., Li L., Wang H., Zhang Y., Cheng X., Zhou N., Rahaman M.N., Liu Z., Huang W., Zhang C. (2015). Wound dressings composed of copper-doped borate bioactive glass microfibers stimulate angiogenesis and heal full-thickness skin defects in a rodent model. Biomaterials.

[43] Balasubramanian P., Hupa L., Jokic B., Detsch R., Grünewald A., Boccaccini A.R. (2016). Angiogenic potential of boron-containing bioactive glasses: In Vitro study. J. Mater. Sci..

[44] Naseri S., Lepry W.C., Nazhat S.N. (2017). Bioactive glasses in wound healing: Hope or hype?. J. Mater. Chem. B.

[45] Ottomeyer M., Mohammadkah A., Day D., Westenberg D. (2016). Broad-Spectrum Antibacterial Characteristics of Four Novel Borate-Based Bioactive Glasses. Adv. Microbiol..

[46] Rahaman M.N., Day D.E., Bal B.S., Fu Q., Jung S.B., Bonewald L.F., Tomsia A.P. (2011). Bioactive glass in tissue engineering. Acta Biomater..

[47] Ding H., Zhao C.-J., Cui X., Gu Y.-F., Jia W.-T., Rahaman M.N., Wang Y., Huang W.-H., Zhang C.-Q. (2014). A Novel Injectable Borate Bioactive Glass Cement as an Antibiotic Delivery Vehicle for Treating Osteomyelitis. PLoS ONE.

[48] Hulsen D.J., van Gestel N.A., Geurts J.A.P., Arts J.J. (2017). S53P4 bioactive glass. Management of Periprosthetic Joint Infections (PJIs).

[49] Liu X., Rahaman M.N., Day D.E. (2013). Conversion of melt-derived microfibrous borate (13–93B3) and silicate (45S5) bioactive glass in a simulated body fluid. J. Mater. Sci. Mater. Med..

[50] Krishnan V., Lakshmi T. (2013). Bioglass: A novel biocompatible innovation. J. Adv. Pharm. Technol. Res..

[51] Coraça-Huber D.C., Fille M., Hausdorfer J., Putzer D., Nogler M. (2014). Efficacy of antibacterial bioactive glass S53P4 againstS. aureusbiofilms grown on titanium discs in vitro. J. Orthop. Res..

[52] Drago L., Romanò D., De Vecchi E., Vassena C., Logoluso N., Mattina R., Romano C.L. (2013). Bioactive glass BAG-S53P4 for the adjunctive treatment of chronic osteomyelitis of the long bones: An in vitroand prospective clinical study. BMC Infect. Dis..

[53] Gergely I., Zazgyva A., Man A., Zuh S., Pop T.S. (2014). The in vitro antibacterial effect of S53P4 bioactive glass and gentamicin impregnated polymethylmethacrylate beads. Acta Microbiol. Immunol. Hung..

[54] Bigoni M., Turati M., Zanchi N., Lombardo A.S., Graci J., Omeljaniuk R.J., Zatti G., Gaddi D. (2019). Clinical applications of Bioactive glass S53P4 in bone infections: A systematic review. Eur. Rev. Med. Pharmacol. Sci..

[55] Miola M., Vitale-Brovarone C., Mattu C., Verné E. (2013). Antibiotic loading on bioactive glasses and glass-ceramics: An approach to surface modification. J. Biomater. Appl..

[56] Rivadeneira J., Luz G.M., Audisio M.C., Mano J.F., Gorustovich A.A. (2015). Novel antibacterial bioactive glass nanocomposite functionalized with tetracycline hydrochloride. Biomed. Glas..

[57] Dorati R., DeTrizio A., Modena T., Conti B., Benazzo F., Gastaldi G., Genta I. (2017). Biodegradable Scaffolds for Bone Regeneration Combined with Drug-Delivery Systems in Osteomyelitis Therapy. Pharmaceuticals.

[58] Xie Z., Cui X., Zhao C., Huang W., Wang J., Zhang C. (2013). Gentamicin-Loaded Borate Bioactive Glass Eradicates Osteomyelitis Due to Escherichia coli in a Rabbit Model. Antimicrob. Agents Chemother..

[59] Xie Z., Liu X., Jia W., Zhang C., Huang W., Wang J. (2009). Treatment of osteomyelitis and repair of bone defect by degradable bioactive borate glass releasing vancomycin. J. Control. Release.

[60] Mancuso E., Bretcanu O., Marshall M., Dalgarno K.W. (2017). Sensitivity of novel silicate and borate-based glass structures on in vitro bioactivity and degradation behaviour. Ceram. Int..

[61] Liu X., Xie Z., Zhang C., Pan H., Rahaman M.N., Zhang X., Fu Q., Huang W. (2010). Bioactive borate glass scaffolds: In Vitro and in vivo evaluation for use as a drug delivery system in the treatment of bone infection. J. Mater. Sci. Mater. Med..

[62] Huang W., Rahaman M.N., Day D.E., Li Y. (2006). Mechanisms for converting bioactive silicate, borate, and borosilicate glasses to hydroxyapatite in dilute phosphate solution. Phys. Chem. Glas. Eur. J. Glas. Sci. Technol. Part B.

[63] Bahniuk M.S., Pirayesh H., Singh H.D., Nychka J.A., Unsworth L.D. (2012). Bioactive Glass 45S5 Powders: Effect of Synthesis Route and Resultant Surface Chemistry and Crystallinity on Protein Adsorption from Human Plasma. Biointerphases.

[64] Catauro M., Raucci M.G., De Gaetano F., Marotta A. (2004). Antibacterial and bioactive silver-containing Na2O·CaO·2SiO2glass prepared by sol–gel method. J. Mater. Sci. Mater. Med..

[65] Lepry W.C., Naseri S., Nazhat S.N. (2017). Effect of processing parameters on textural and bioactive properties of sol–gel-derived borate glasses. J. Mater. Sci..

[66] Lepry W.C., Nazhat S.N. (2015). Highly Bioactive Sol-Gel-Derived Borate Glasses. Chem. Mater..

[67] Hench L.L., Best S.M. (2013). Ceramics, Glasses, and Glass-Ceramics: Basic Principles. Biomaterials Science: An Introduction to Materials.

[68] Faure J., Drevet R., Lemelle A., Ben Jaber N., Tara A., El Btaouri H., Benhayoune H. (2015). A new sol–gel synthesis of 45S5 bioactive glass using an organic acid as catalyst. Mater. Sci. Eng. C.

[69] Pirayesh H., Nychka J.A. (2013). Sol-Gel Synthesis of Bioactive Glass-Ceramic 45S5 and its in vitro Dissolution and Mineralization Behavior. J. Am. Ceram. Soc..

[70] Li R., Clark A.E., Hench L.L. (1991). An investigation of bioactive glass powders by sol-gel processing. J. Appl. Biomater..

[71] Vallet-Regí M., Salinas A.J., Arcos D. (2016). Tailoring the Structure of Bioactive Glasses: From the Nanoscale to Macroporous Scaffolds. Int. J. Appl. Glas. Sci..

[72] Gmeiner R., Deisinger U., Schönherr J., Lechner B., Detsch R., Boccaccini A.R., Stampfl J. (2015). Additive manufacturing of bioactive glasses and silicate bioceramics. J. Ceram. Sci. Technol..

[73] Cho J., Cannio M., Boccaccini A.R. (2009). The Electrophoretic Deposition of Bioglass^®^/Carbon Nanotube composite layers for bioactive coatings. Int. J. Mater. Prod. Technol..

[74] Eqtesadi S., Motealleh A., Miranda P., Pajares A., Lemos A., Ferreira J. (2014). Robocasting of 45S5 bioactive glass scaffolds for bone tissue engineering. J. Eur. Ceram. Soc..

[75] Tesavibul P., Felzmann R., Gruber S., Liska R., Thompson I., Boccaccini A.R., Stampfl J. (2012). Processing of 45S5 Bioglass^®^ by lithography-based additive manufacturing. Mater. Lett..

[76] Boccaccini D., Cannio M., Bernardo E., Boccaccini A.R. (2021). Glass and Glass-Ceramic Matrix Composites for Advanced Applications: Part I: Properties and Manufacturing Technologies. Encyclopedia of Materials: Technical Ceramics and Glasses.

[77] Furlan R.G., Correr W.R., Russi A.F.C., Iemma M.R.D.C., Trovatti E., Pecoraro É. (2018). Preparation and characterization of boron-based bioglass by sol−gel process. J. Sol. Gel Sci. Technol..

[78] Zheng K., Boccaccini A.R. (2017). Sol-gel processing of bioactive glass nanoparticles: A review. Adv. Colloid Interface Sci..

[79] Wang X., Zhang Y., Lin C., Zhong W. (2017). Sol-gel derived terbium-containing mesoporous bioactive glasses nanospheres: In Vitro hydroxyapatite formation and drug delivery. Colloids Surf. B Biointerfaces.

[80] Siaili M., Chatzopoulou D., Gillam D. (2018). An overview of periodontal regenerative procedures for the general dental practitioner. Saudi Dent. J..

[81] Huygh A., Schepers E.J.G., Barbier L., Ducheyne P. (2002). Microchemical transformation of bioactive glass particles of narrow size range, a 0–24 months study. J. Mater. Sci. Mater. Med..

[82] Wilson J., Low S.B. (1992). Bioactive ceramics for periodontal treatment: Comparative studies in the patus monkey. J. Appl. Biomater..

[83] Wilson J., Merwin G.E. (1988). Biomaterials for facial bone augmentation: Comparative studies. J. Biomed. Mater. Res..

[84] Zamet J.S., Darbar U.R., Griffiths G., Bulman J., Brägger U., Burgin W., Newman H.N. (1997). Particulate bioglassR as a grafting material in the treatment of periodontal intrabony defects. J. Clin. Periodontol..

[85] Low S.B., King C.J., Krieger J. (1997). An evaluation of bioactive ceramic in the treatment of periodontal osseous defects. Int. J. Periodontics Restor. Dent..

[86] Mengel R., Schreiber D., Flores-De-Jacoby L. (2006). Bioabsorbable Membrane and Bioactive Glass in the Treatment of Intrabony Defects in Patients with Generalized Aggressive Periodontitis: Results of a 5-Year Clinical and Radiological Study. J. Periodontol..

[87] Gatti A.M., Simonetti L.A., Monari E., Guidi S., Greenspan D. (2006). Bone Augmentation with Bioactive Glass in Three Cases of Dental Implant Placement. J. Biomater. Appl..

[88] Banerjee A., Hajatdoost-Sani M., Farrell S., Thompson I. (2010). A clinical evaluation and comparison of bioactive glass and sodium bicarbonate air-polishing powders. J. Dent..

[89] Yadav V.S., Narula S., Sharma R., Tewari S., Yadav R. (2011). Clinical evaluation of guided tissue regeneration combined with autogenous bone or autogenous bone mixed with bioactive glass in intrabony defects. J. Oral Sci..

[90] Subbaiah R., Thomas B. (2011). Efficacy of a bioactive alloplast, in the treatment of human periodontal osseous defects-a clinical study. Med. Oral Patol. Oral Cir. Bucal.

[91] Stavropoulos A., Sima C., Sima A., Nyengaard J., Karring T., Sculean A. (2012). Histological evaluation of healing after transalveolar maxillary sinus augmentation with bioglass and autogenous bone. Clin. Oral Implant. Res..

[92] Sohrabi K., Saraiya V., Laage T.A., Harris M., Blieden M., Karimbux N. (2012). An Evaluation of Bioactive Glass in the Treatment of Periodontal Defects: A Meta-Analysis of Randomized Controlled Clinical Trials. J. Periodontol..

[93] Chacko N.L., Abraham S., Rao H.N.S., Sridhar N., Moon N., Barde D.H. (2014). A Clinical and Radiographic Evaluation of Periodontal Regenerative Potential of PerioGlas^®^: A Synthetic, Resorbable Material in Treating Periodontal Infrabony Defects. J. Int. Oral Health.

[94] Pereira R.D.S., Menezes J.D., Bonardi J.P., Griza G.L., Okamoto R., Hochuli-Vieira E. (2017). Histomorphometric and immunohistochemical assessment of RUNX2 and VEGF of Biogran™ and autogenous bone graft in human maxillary sinus bone augmentation: A prospective and randomized study. Clin. Implant. Dent. Relat. Res..

[95] Pereira R., Menezes J., Bonardi J., Griza G., Okamoto R., Hochuli-Vieira E. (2017). Comparative study of volumetric changes and trabecular microarchitecture in human maxillary sinus bone augmentation with bioactive glass and autogenous bone graft: A prospective and randomized assessment. Int. J. Oral Maxillofac. Surg..

[96] Lovelace T.B., Mellonig J.T., Meffert R.M., Jones A.A., Nummikoski P.V., Cochran D.L. (1998). Clinical Evaluation of Bioactive Glass in the Treatment of Periodontal Osseous Defects in Humans. J. Periodontol..

[97] Froum S.J., Weinberg M.A., Tarnow D. (1998). Comparison of Bioactive Glass Synthetic Bone Graft Particles and Open Debridement in the Treatment of Human Periodontal Defects. A Clinical Study. J. Periodontol..

[98] Tadjoedin E.S., Lange G.L., Holzmann P.J., Kuiper L., Burger E.H. (2000). Histological observations on biopsies harvested following sinus floor elevation using a bioactive glass material of narrow size range. Clin. Oral Implant. Res..

[99] Nevins M., Marcelo M., Nevins V., King C.J., Mscd D.D.S., Dds D.M.S.V., Schenk R.K., Fiorellini V.P. (2000). Human Histologie Evaluation of Bioactive Ceramic in the Treatment of Periodontai Osseous Defects. Int J. Periodontics Restor. Dent..

[100] Cordioli G., Mazzocco C., Schepers E., Brugnolo E., Majzoub Z. (2001). Maxillary sinus floor augmentation using bioactive glass granules and autogenous bone with simultaneous implant placement. Clin. Oral Implant. Res..

[101] Mengel R., Soffner M., Flores-De-Jacoby L. (2003). Bioabsorbable Membrane and Bioactive Glass in the Treatment of Intrabony Defects in Patients with Generalized Aggressive Periodontitis: Results of a 12-Month Clinical and Radiological Study. J. Periodontol..

[102] El-Ghannam A., Amin H., Nasr T., Shama A. (2004). Enhancement of bone regeneration and graft material resorption using surface-modified bioactive glass in cortical and human maxillary cystic bone defects. Int. J. Oral Maxillofac. Implant..

[103] Sculean A., Barbé G., Chiantella G.C., Arweiler N.B., Berakdar M., Brecx M. (2002). Clinical Evaluation of an Enamel Matrix Protein Derivative Combined With a Bioactive Glass for the Treatment of Intrabony Periodontal Defects in Humans. J. Periodontol..

[104] Hench L.L., Wilson J. (1993). An Introduction to Bioceramics. World Sci..

[105] Ratner B.D., Hoffman A.S., Schoen F.J., Lemons J.E. (2004). Biomaterials Science: An Introduction to Materials in Medicine.

[106] Boyne P.J., James R.A. (1980). Grafting of the maxillary sinus floor with autogenous marrow and bone. J. Oral Surg..

[107] Lindfors N.C. (2011). Clinical Experience on Bioactive Glass S53P4 in Reconstructive Surgery in the Upper Extremity Showing Bone Remodelling, Vascularization, Cartilage Repair. J. Biotechnol. Biomater..

[108] Pernaa K., Koski I., Mattila K., Gullichsen E., Heikkila J., Aho A., Lindfors N. (2011). Bioactive glass S53P4 and autograft bone in treatment of depressed tibial plateau fractures—A prospective randomized 11-year follow-up. J. Long. Term. Eff. Med. Implants.

[109] Heikkilä J.T., Kukkonen J., Aho A.J., Moisander S., Kyyrönen T., Mattila K. (2011). Bioactive glass granules: A suitable bone substitute material in the operative treatment of depressed lateral tibial plateau fractures: A prospective, randomized 1 year follow-up study. J. Mater. Sci. Mater. Med..

[110] Frantzén J., Rantakokko J., Aro H.T., Heinänen J., Kajander S., Gullichsen E., Kotilainen E., Lindfors N.C. (2011). Instrumented spondylodesis in degenerative spondylolisthesis with bioactive glass and autologous bone: A prospective 11-year follow-up. J. Spinal Disord. Tech..

[111] Rantakokko J., Frantzén J.P., Heinänen J., Kajander S., Kotilainen E., Gullichsen E., Lindfors N. (2012). Posterolateral Spondylodesis Using Bioactive Glass S53P4 and Autogenous Bone in Instrumented Unstable Lumbar Spine Burst Fractures. Scand. J. Surg..

[112] Sarin J., Grénman R., Aitasalo K., Pulkkinen J. (2012). Bioactive Glass S53P4 in Mastoid Obliteration Surgery for Chronic Otitis Media and Cerebrospinal Fluid Leakage. Ann. Otol. Rhinol. Laryngol..

[113] Silvola J.T. (2012). Mastoidectomy cavity obliteration with bioactive glass: A pilot study. Otolaryngol. Head Neck Surg..

[114] McAndrew J., Efrimescu C., Sheehan E., Niall D. (2013). Through the looking glass; bioactive glass S53P4 (BonAlive^®^) in the treatment of chronic osteomyelitis. Ir. J. Med Sci..

[115] Romanò C.L., Logoluso N., Meani E., Romanò D., De Vecchi E., Vassena C., Drago L. (2014). A comparative study of the use of bioactive glass S53P4 and antibiotic-loaded calcium-based bone substitutes in the treatment of chronic osteomyelitis: A retrospective comparative study. Bone Jt. J..

[116] Stoor P., Mesimäki K., Lindqvist C., Kontio R. (2015). The use of anatomically drop-shaped bioactive glass S53P4 implants in the reconstruction of orbital floor fractures—A prospective long-term follow-up study. J. Cranio-Maxillofac. Surg..

[117] Kinnunen I., Aitasalo K., Pöllönen M., Varpula M. (2000). Reconstruction of orbital floor fractures using bioactive glass. J. Cranio-Maxillofac. Surg..

[118] Piitulainen J.M., Posti J.P., Aitasalo K.M.J., Vuorinen V., Vallittu P.K., Serlo W. (2015). Paediatric cranial defect reconstruction using bioactive fibre-reinforced composite implant: Early outcomes. Acta Neurochir..

[119] Aurégan J.-C., Bégué T. (2015). Bioactive glass for long bone infection: A systematic review. Injury.

[120] Schimanski G., Schimanski E. (2015). [Obliteration of mastoid cavities: 30 years of experience with recommendations for surgical strategy]. HNO.

[121] Kankare J., Lindfors N.C. (2016). Reconstruction of Vertebral Bone Defects using an Expandable Replacement Device and Bioactive Glass S53P4 in the Treatment of Vertebral Osteomyelitis: Three Patients and Three Pathogens. Scand. J. Surg..

[122] Bernardeschi D., Pyatigorskaya N., Russo F.Y., De Seta D., Corallo G., Ferrary E., Nguyen Y., Sterkers O. (2017). Anatomical, functional and quality-of-life results for mastoid and epitympanic obliteration with bioactive glass s53p4: A prospective clinical study. Clin. Otolaryngol..

[123] De Vej Mestdagh P.D., Colnot D.R., Borggreven P.A., Orelio C.C., Quak J.J. (2017). Mastoid obliteration with S53P4 bioactive glass in cholesteatoma surgery. Acta Otolaryngol..

[124] Vos J., De Vej Mestdagh P.D., Colnot D., Borggreven P., Orelio C., Quak J. (2017). Bioactive glass obliteration of the mastoid significantly improves surgical outcome in non-cholesteatomatous chronic otitis media patients. Eur. Arch. Oto-Rhino-Laryngol..

[125] Stoor P., Apajalahti S., Kontio R. (2017). Regeneration of Cystic Bone Cavities and Bone Defects with Bioactive Glass S53P4 in the Upper and Lower Jaws. J. Craniofacial Surg..

[126] Stoor P., Apajalahti S. (2017). Osteotomy Site Grafting in Bilateral Sagittal Split Surgery With Bioactive Glass S53P4 for Skeletal Stability. J. Craniofacial Surg..

[127] Syvänen J., Nietosvaara Y., Kohonen I., Koskimies E., Haara M., Korhonen J., Pajulo O., Helenius I. (2017). Treatment of Aneurysmal Bone Cysts with Bioactive Glass in Children. Scand. J. Surg..

[128] Aitasalo K., Kinnunen I., Palmgren J., Varpula M. (2001). Repair of orbital floor fractures with bioactive glass implants. J. Oral Maxillofac. Surg..

[129] Turunen T., Peltola J., Yli-Urpo A., Happonen R.-P. (2004). Bioactive glass granules as a bone adjunctive material in maxillary sinus floor augmentation. Clin. Oral Implant. Res..

[130] Peltola M., Aitasalo K., Suonpää J., Varpula M., Yli-Urpo A. (2006). Bioactive glass S53P4 in frontal sinus obliteration: A long-term clinical experience. Head Neck.

[131] Peltola M.J., Aitasalo K.M., Aho A.J., Tirri T., Suonpää J.T. (2008). Long-Term Microscopic and Tissue Analytical Findings for 2 Frontal Sinus Obliteration Materials. J. Oral Maxillofac. Surg..

[132] Peltola M., Kinnunen I., Aitasalo K. (2008). Reconstruction of Orbital Wall Defects With Bioactive Glass Plates. J. Oral Maxillofac. Surg..

[133] Lindfors N., Hyvönen P., Nyyssönen M., Kirjavainen M., Kankare J., Gullichsen E., Salo J. (2010). Bioactive glass S53P4 as bone graft substitute in treatment of osteomyelitis. Bone.

[134] Lindfors N.C., Koski I., Heikkilä J.T., Mattila K., Aho A.J. (2010). A prospective randomized 14-year follow-up study of bioactive glass and autogenous bone as bone graft substitutes in benign bone tumors. J. Biomed. Mater. Res. Part. B Appl. Biomater..

[135] Balasubramanian P., Büttner T., Pacheco V.M., Boccaccini A.R. (2018). Boron-containing bioactive glasses in bone and soft tissue engineering. J. Eur. Ceram. Soc..

[136] Abdelghany A. (2013). Novel method for early investigation of bioactivity in different borate bio-glasses. Spectrochim. Acta Part A Mol. Biomol. Spectrosc..

[137] Richard M.N. (2000). Bioactive Behavior of a Borate Glass. Ph.D. Thesis.

[138] Fu Q., Rahaman M.N., Fu H., Liu X. (2010). Silicate, borosilicate, and borate bioactive glass scaffolds with controllable degradation rate for bone tissue engineering applications. I. Preparation and in vitro degradation. J. Biomed. Mater. Res. Part A.

[139] Deliormanlı A.M., Al-Buriahi M.S., Somaily H.H., Tekin H.O. (2021). 13-93B3 Bioactive glasses containing Ce^3+^, Ga^3+^ and V^5+^: Dose rate and gamma radiation characteristic for medical purposes. Appl. Phys. A Mater. Sci. Process..

[140] Liu X., Huang W., Fu H., Yao A., Wang D., Pan H., Lu W.W., Jiang X., Zhang X. (2009). Bioactive borosilicate glass scaffolds: In vitro degradation and bioactivity behaviors. J. Mater. Sci. Mater. Med..

[141] Arango-Ospina M., Hupa L., Boccaccini A.R. (2019). Bioactivity and dissolution behavior of boron-containing bioactive glasses under static and dynamic conditions in different media. Biomed. Glas..

[142] Lepry W.C., Nazhat S.N. (2021). A Review of Phosphate and Borate Sol–Gel Glasses for Biomedical Applications. Adv. NanoBiomed Res..

[143] Xia L., Ma W., Zhou Y., Gui Z., Yao A., Wang D., Takemura A., Uemura M., Lin K., Xu Y. (2019). Stimulatory Effects of Boron Containing Bioactive Glass on Osteogenesis and Angiogenesis of Polycaprolactone: In Vitro Study. BioMed Res. Int..

[144] Zhang P., Yang K., Zhou Z., Zhu X., Li W., Cao C., Zhou K., Liao L., Ai F. (2020). Customized Borosilicate Bioglass Scaffolds With Excellent Biodegradation and Osteogenesis for Mandible Reconstruction. Front. Bioeng. Biotechnol..

[145] Zheng K., Fan Y., Torre E., Balasubramanian P., Taccardi N., Cassinelli C., Morra M., Iviglia G., Boccaccini A.R. (2020). Incorporation of Boron in Mesoporous Bioactive Glass Nanoparticles Reduces Inflammatory Response and Delays Osteogenic Differentiation. Part. Part. Syst. Charact..

[146] Naseri S., Nazhat S.N. (2017). 14—Bioactive and soluble glasses for wound-healing applications. Bioactive Glasses: Materials, Properties and Applications.

[147] Jung S. (2010). Borate Based Bioactive Glass Scaffolds for Hard and Soft Tissue Engineering. Ph.D. Thesis.

[148] Wray P. (2010). “Cotton Candy” That Heals?. Am. Ceram. Soc. Bull..

[149] Liu X., Rahaman M.N., Hilmas G., Bal B.S. (2013). Mechanical properties of bioactive glass (13-93) scaffolds fabricated by robotic deposition for structural bone repair. Acta Biomater..

[150] Cannillo V., Leonelli C., Boccaccini A.R. (2002). Numerical models for thermal residual stresses in Al2O3 platelets/borosilicate glass matrix composites. Mater. Sci. Eng. A.

[151] Cannillo V., Montorsi M., Siligardi C., Sola A., De Portu G., Micele L., Pezzotti G. (2006). Microscale computational simulation and experimental measurement of thermal residual stresses in glass–alumina functionally graded materials. J. Eur. Ceram. Soc..

[152] Cattini A., Łatka L., Bellucci D., Bolelli G., Sola A., Lusvarghi L., Pawłowski L., Cannillo V. (2013). Suspension plasma sprayed bioactive glass coatings: Effects of processing on microstructure, mechanical properties and in-vitro behaviour. Surf. Coat. Technol..

[153] Cannillo V., Colmenares-Angulo J., Lusvarghi L., Pierli F., Sampath S. (2009). In vitro characterisation of plasma-sprayed apatite/wollastonite glass–ceramic biocoatings on titanium alloys. J. Eur. Ceram. Soc..

